# Fabrication of Optical Fibers with Multiple Coatings for Swelling-Based Chemical Sensing

**DOI:** 10.3390/mi12080941

**Published:** 2021-08-10

**Authors:** Dorel Dorobantu, Alin Jderu, Marius Enachescu, Dominik Ziegler

**Affiliations:** 1 Center for Surface Science and Nanotechnology (CSSNT), University of Politehnica Bucharest, 060042 Bucharest, Romania; dorel.dorobantu@cssnt-upb.ro (D.D.); alin.jderu@cssnt-upb.ro (A.J.); marius.enachescu@cssnt-upb.ro (M.E.); 2 SC NanoPRO START S.R.L., Oltenitei, No. 388, District 4, 041337 Bucharest, Romania; 3 Academy of Romanian Scientists, 54 Splaiul Independentei, 050094 Bucharest, Romania

**Keywords:** chemical sensing, optical fibers, coatings, microfabrication

## Abstract

We discuss distributed chemical sensing based on the swelling of coatings of optical fibers. Volume changes in the coating induce strain in the fiber’s glass core, provoking a local change in the refractive index which is detectable by distributed fiber optical sensing techniques. We describe methods to realize different coatings on a single fiber. Simultaneous detection of swelling processes all along the fiber opens the possibility to interrogate thousands of differently functionalized sections on a single fiber. Principal component analysis is used to enable sensors for environmental monitoring, food analysis, agriculture, water quality monitoring, or medical diagnostics.

## 1. Introduction

Optical fibers, with their high bandwidth, high reliability, low losses, and low cost, are the backbone of today’s telecommunications networks that reach data throughputs above one petabit per second [1]. Immunity to electromagnetic fields and remarkable light guidance make optical fiber also an extraordinary element for sensing applications. Among the different fiber-optic sensing approaches, fiber Bragg gratings (FBG) sensors [2,3] are one of the most mature technologies. However, they typically only provide information only from the location where the grating is inscribed. On the contrary, distributed optical fiber sensors [4] have obtained great attention over recent decades due to their unique feature to provide spatially resolved and longitudinally distributed measurements of physical variables, such as temperature or strain, along the entire length of an unmodified telecom fiber. Fiber interrogators typically use optical time-domain reflectometry (OTDR) or optical frequency-domain reflectometry (OFDR) to obtain spatially resolved information about the measurand. OTDR is commonly used to reach very long sensing distances; however, it provides limited spatial resolution. On the other hand, OFDR is exploited to achieve very high spatial resolution (e.g., millimetric scale), although it can be restricted to applications that require only short sensing distances (few tens of meters). The recent advances on distributed optical fiber sensing techniques lead to a significant increase in the number of possible fiber sensing applications [5]. Applications of distributed sensing range from structural health monitoring and shape sensing, to pipeline and electrical transmission line monitoring, or intrusion detection for perimeter security applications [6].

Optical fiber approaches have been investigated for many years for chemical sensing. The methods can be classified as absorbance-based sensors, interferometric sensors, fluorescence-based sensors, fiber grating sensors, or resonance-based sensors [7]. Silica optical fibers are chemically inert, hence, fiber-optic based chemical sensing commonly requires the transduction of the signal of interest into changes of temperature, strain, or optical power loss. For this reason, the majority of approaches only probe the chemical environment at the end of the optical fiber or at few discrete locations where the light is purposefully guided into a sensing element [8,9,10]. In the case of distributed chemical sensing [11], polymer optical fibers or microstructured fibers are typically used. For instance, microstructured fibers in combination with OTDR or OFDR interrogation can allow for highly sensitive distributed gas sensing [12,13]. On the other hand, plastic optical fibers are inherently sensitive to humidity, resulting in several distributed humidity sensors that have been demonstrated based on Rayleigh or Brillouin scattering and different types of polymer fibers. Using water-swellable polymers to coat silica fibers and reflectometry approaches, distributed humidity sensors over conventional glass fibers have also been reported [8]. The coating of the optical fiber can be a specific material that allows for specific chemical sensing [14,15,16]. While this approach has been mostly used for point sensors [17], where several possible coatings for chemical sensing have been investigated [17,18,19], we have shown that the approach can be directly applied to distributed sensing approaches [16].

**Figure 1:**
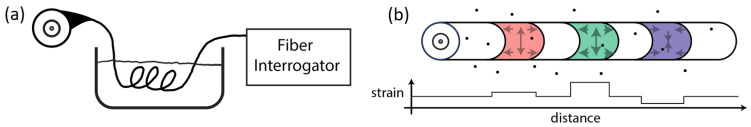
(**a**) Principle of swelling-based chemical sensing: an optical fiber with a polymeric coating is immersed in an analyte and a fiber interrogator is used to monitor the strain transduced to the silica core. (**b**) A fiber can contain segments with different coating materials which all can be read out simultaneously using distributed sensing. Exposure to an analyte (illustrated by the black dots) leads to different swelling of the distinct coating materials (red, green, purple) such that a “fingerprint” strain signature of the analyte can be recorded simultaneously.

In this work, we investigate fabrication methods to realize optical fibers which have multiple coatings and therefore can be used for more advanced chemical sensing. As shown in Figure 1a, an optical fiber is immersed in an analyte and a fiber optical interrogator is used to detect the strain induced by the swelling. The concept of using multiple coatings enables new sensing approaches. As illustrated in Figure 1b, swelling of the fiber’s coating is converted into radial and longitudinal strain, the latter induces a local refractive index change in the optical fiber.

For the fabrication of chemical sensors, the choice of a swelling material is crucial; factors influencing the choice are the cross-sensitivity and manufacturability [20]. An ideal coating material must show a high swelling and at the same time high Young’s modulus to build up large strains. The range of suitable coating for chemical sensing depends on the mechanical stability, thermal response, and physicochemical response of the used materials. We demonstrate, with a few simple fabrication methods, that simultaneous detection of the response of different coatings is possible thanks to distributed sensing.

## 2. Materials and Methods

### 2.1. Realization of Multiple Sensor on a Single Fiber

Commercial optical fibers were produced with a polymeric coating to protect and mechanical strength was provided to the optical waveguide that is made out of silica. In current practice, most fiber manufactures use a dual-layer coating technique. The primary coating was rubbery and served as a cushion to protect the glass fiber from mechanical loads. The second stiffer coating layer protected the fiber from abrasions and environmental exposure. Both layers were typically urethane- or acrylate-based but could contain photo initiators and additives, whose exact composition is a trade secret of the manufacturers. For this work, fibers we employed were single-mode optical fibers manufactured by Corning, type Corning SMF-28, core diameter: 8.3 µm, cladding diameter; 125 µm, coating diameter; and 245 µm, coating-cladding concentricity <12 µm. The coatings were applied sequentially in liquid form and are cured by exposure to ultraviolet light. We demonstrated that coatings on commercially available fibers could be used for chemical sensing [16]. Here, we investigated methods with which different sensing elements can be realized on the same optical fiber which has multiple sections with different chemistries.

### 2.2. Fusion Splicing of Differently Coated Fibers

**Figure 2:**
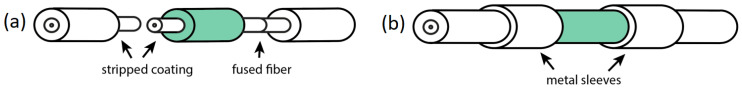
(**a**) For fusion splicing the coating is stripped off the ends of two types of fibers and an electric arc is used to melt the fiber’s core and cladding back together (**b**) to increase mechanical stability and protect the fused bare fibers metal sleeves are applied over the junctions.

The easiest approach to realize a single fiber with different coatings was to join two coated fibers with different composition. Fusion splicing was the standard method for joining two cleanly cut fiber ends. As illustrated in Figure 2a, the coating was first stripped away from the last few millimeters of the fiber, such that only the glass materials (i.e., the cladding and core) remain. Modern fusion splicing tools automatically aligned the two stripped ends before applying an electric arc to fuse them together. After fusion, only insignificant optical losses on the order of 0.1 dB or smaller were observed. However, the fused fiber optic section was prone to fracturing, and the breaking load under tension was smaller. A cost-effective reliable mechanical protection were metal sleeves, whereby a steel rod provided rigid support for the splice joint and an outer shrink tube holds the metal tube through which the fiber was threaded (See Figure 2b). This provided a replacement for the original fiber coating and prevented the spliced area from bending.

### 2.3. Testing of the Strain Response in Water and Water/Ethanol Mixtures

To test the swelling of the fiber assembly with different sections, we exposed the fiber to water and ethanol mixtures while continuously monitoring the Rayleigh scattering in the fiber. A commercial OFDR system was used for this purpose (ODiSI-B by Luna Technologies Inc., Roanoke, VA, USA). This system provided a spatial resolution of 1.25 mm and 4 Hz sampling rate. After mounting the fiber in the empty bath, we first measured the baseline strain in air for 5 min. Then, we gently filled the bath with water and recorded the evolution of the strain over 10 min. Every 10 min we exchanged the liquids in the following sequence: water, 20%v EtOH, water, 40%v EtOH, water, 60%v EtOH. For exchanging liquids, a syringe was used to remove a fixed amount of the existing liquid from the bath and the required amount to reach target concentrations was quickly replenished. Since ethanol is a polar solvent readily miscible in water, no stirring was required to achieve uniform concentrations throughout the bath.

**Figure 3:**
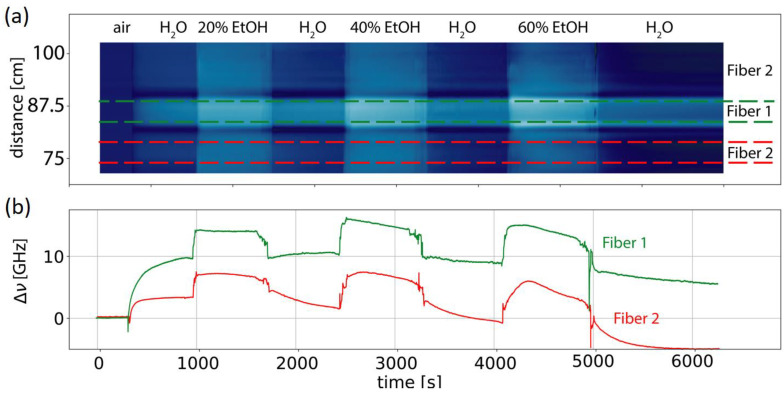
(**a**) Colormap of the observed optical frequency shift (∆ν) against distance and time. (**b**) Rayleigh frequency shifts averaged over the different fiber segments as indicated in the top graph. Each distance increment corresponds to 1.25 mm.

Figure 3 shows the observed Rayleigh frequency shift (∆ν) for an about 30 cm long fiber that contains segments with the two coatings. Clearly, one observed a different response for the two fibers when exposed to water. Over the length of the metal sleeves, the strain remained low and close to the pre-recorded values in air. Both fibers showed a similar increase in strain when exposed to ethanol–water mixtures. However, when reverting back to water after exposure to the first ethanol-water mixture (20 volume percent), fiber 1 maintained the water strain levels while fiber 2 did not. This can be evidence of delamination or partial dissolution of the coating material. As discussed in the following, we discuss methods to recoat fiber with different coating materials.

### 2.4. Methods to Recoat Optical Fibers with Multiple Coatings

#### 2.4.1. Removing of the Original Coating

The first step towards recoating segments of a single optical fiber with coatings of different chemical composition was to strip off the original coating. Thermo-mechanically stripping, reactive ion plasma etching, laser ablation, or dissolution using dichloromethane or hot sulfuric acid [21] were demonstrated. We experimented with dichloromethane; however, for the fibers used in this work, only a partial dissolution was observed even after a prolonged exposure of >30 min. The residual coating could only be removed by rubbing, which increased the risk of fracturing the silica fiber. Moreover, chemical dissolution results in poorly defined edges of the coating. Although, reactive ion etching [22] and laser machining [23] were demonstrated to remove coating, one of the simplest method for removing the coating was mechanical stripping of the fiber coating. To this end, a fiber stripping tool with a hole and a diameter which matched the cladding of the fiber such that the coating could be stripped away easily, while exposing the cladding fiber. With some practice, it became possible to only strip a segment with a length of about millimeter to few centimeters without damaging the cladding fiber and the fiber core.

#### 2.4.2. Molding of UV Crosslinking Polymer

**Figure 4:**
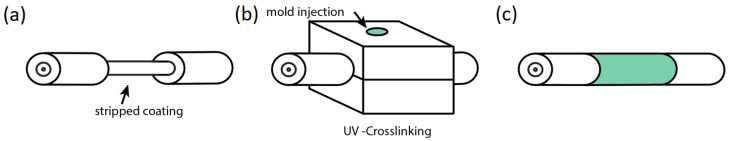
Steps to recoat optical fibers using a fiber recoater. (**a**) Stripping of polymer coating; (**b**) Polymer injection through a channel in the top plate into the mold assembly; (**c**) Recoated fiber via injection.

Molding is compatible with materials that crosslink, either through spontaneous or photoinduced chemical reactions. A conventional method to recoat fibers was to use fiber recoaters which offer dedicated mold assemblies and ultraviolet (UV) light sources to crosslink photopolymers. To test this method, we used on a manual recoater with a hinged top that could be opened and closed by hand (Vytran, PTR200-MRR). We used a low-index UV-cured fluoropolymer (EFIRON SPC-373AP; Fospia Co., Ltd., Gyeonggi-do, Korea). As shown in Figure 4, the polymer was either injected through a channel in the top plate into the mold assembly, or a droplet was applied around the fiber prior to closing the mold. The injection method was preferred as it gave better control of the volume and avoids air inclusions as the polymer pushes out air from the middle to both open ends of the mold. Exposure with the integrated UV lamp crosslinked the polymer. Compared to the metal sleeves, fiber recoating not only restored the mechanical strength but also gave the fiber the same flexibility as when originally manufactured.

**Figure 5:**
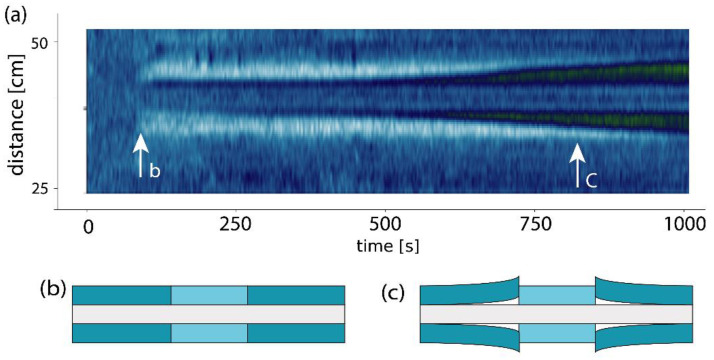
(**a**) For immersion in 80% Acetone the strain sensing can reveal a delamination of the acrylic coating that results in a reduction in the detected strain. As shown in the inset arrows for (**b**) the coating still builds up a strain, but over time the strain decreases. (**c**) illustration how the delamination propagates (at about 100 µm/s) in both directions. The EFIROM coating itself remains intact over the entire time of the measurement but does not change in strain when exposed to any of the tested solvents (water, ethanol, isopropanol and acetone).

The use of OFDR already during the fabrication could help to optimize process parameters during UV-exposure or heating in the curing processes. Moreover, it helped for testing and failure analysis for instance detecting detachment of the coatings. Figure 5a shows a colormap of the Rayleigh frequency shift with the fluoropolymer coated fiber in acetone displayed against time and distance. After about 10 min, the acrylic coating gradually detached from the fiber; over the entire segment that was exposed to ethanol, the same effect was observed with acetone. Such effect was observed with non-stripped fibers [16], or the spliced fibers with metal sleeves. Observation of the fiber before and after the exposure under an optical microscope did not reveal any visible difference. As illustrated in Figure 5b, we hypnotize that the fiber detached or partially dissolved the first rubbery polymer coating as it got exposed by the stripping and recoating of the fiber.

#### 2.4.3. Dip Coating and Paint Coating

While dip coating is seemingly easiest, we observed for many low viscosity solutions that it was not possible to obtain conformal coatings. Due to the surface energy droplet formation occurred, resulting in highly non-conformal coatings or even dripping off of the entire material. However, with higher viscosity materials it was possible to coat the stripped part of the fiber. For instance, we dissolved poly (methyl methacrylate) (PMMA), i.e., shavings from a commercial Plexiglas slab, in dichloromethane. Due to evaporation of the dichloromethane at the liquid–air interface, a thin film started to solidify on the surface. When dipping in the stripped optical fiber, is was easy to transfer PMMA onto the fiber. Alternatively, it was easy to paint coat the stripped fiber part with film-forming polymer that solidified by solvent evaporation and UV curing. We demonstrated this used nitrocellulose, which is frequently used as a film-forming polymer in nail polishing. The solvents used are acetates but solidification of the polymer is not only given by solvent evaporation, but further supported by UV-light-triggered polymerization of benzol peroxides [24]. This fact made it possible to paint coat fibers without the before-mentioned issues of forming small droplets. We applied two sequential coatings to reach a thickness resulting in a diameter of the fibers, i.e., about 250 µm. Independent of the recoating procedure, we ended up with as many sensors as designed by as many exposures of the fiber cladding, implemented after mechanically stripping of the fiber coating. Upon exposure to an analyte, the recoating material swelled if the analyte molecules could diffuse into the matrix of the recoated material. Swelling means a volume change in the recoating that can happen radially and axially. The axial swelling induced a strain in the fiber’s glass core, provoking a local change in the refractive index which was detectable by distributed fiber optical sensing techniques, e.g., OFDR.3.3 Verification by strain sensing from different chemistries coated on a single fiber.

**Figure 6:**
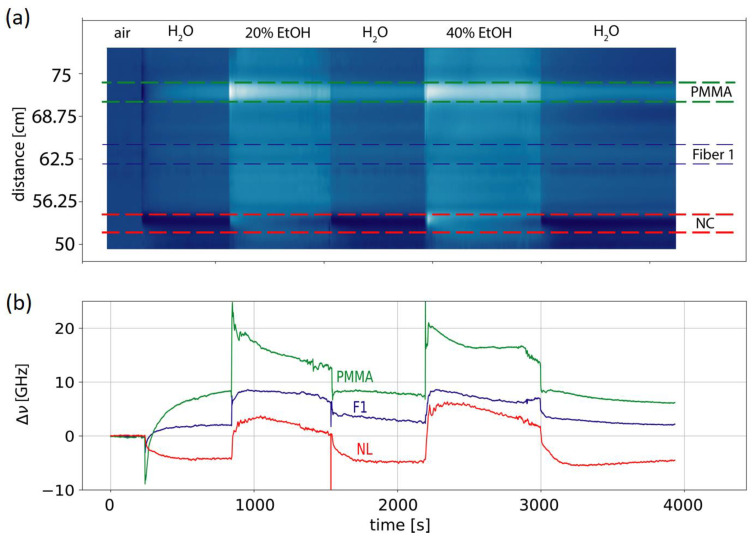
(**a**) Colormap showing the observed Rayleigh frequency shifts for a fiber with 3 different coatings (Original coating (ORG), polymethyl methacrylate (PMMA) and nitrocellulose (NC) coatings). A baseline was first recorded in air then the fiber is exposed for approximately 10 min long periods to water followed by 20% ethanol, water, 40% ethanol, and water as indicated. (**b**) Average frequency shifts for zones with different coatings as indicated with the dashed lines.

Lastly, to test the coatings and demonstrate that multiple different chemistries could be read out simultaneously, we modified a common fiber with two approximately 2-cm-long sections with PMMA and nitrocellulose coating. As shown in Figure 6, the coated sections showed a different response to water and ethanol. The colormap shows the observed Rayleigh frequency shifts for a fiber with 3 different coatings: original coating (ORG), polymethyl methacrylate (PMMA), and nitrocellulose (NC) coatings. A baseline was first recorded in air, then the fiber was exposed for approximately 10-min-long periods to water, followed by 20% ethanol, water, 40% ethanol, and water, as indicated. The average frequency shifted for zones with different coatings (as indicated by the dashed lines).

More information might have been derived when including the temporal evolution of the strain. We have shown that, in the first few minutes, there can be thermal effects due to heating by the enthalpy of mixing. However, they can be excluded when uncoated fiber segments are used to measure the temperature independently [16]. This preliminary result shows that arrays of differently coated sensors materials can be fabricated and read out, simultaneously. Certainly, the repeatability and the reliability of such sensors needs more work and more experiments. In our experiments, e.g., Figure 6, the repeatability of using recoated sensors for measurements in water and EtOH was already demonstrated. This work highlights the repeatability and reliability for only a few such cycles. However, in the lab, we demonstrated this repeatability for much higher numbers of such cycles of repeated exposure of the recoated sensor to different analytes. We expect the recoated materials to be influenced by the time/aging and environment. However, we consider it very likely for such a recoated material to still react to analytes, even with a lower amplitude of the swelling/signal, which will still be detectable using the distributed sensing approach. Naturally, such dedicated experiments should be performed.

## 3. Discussion

The preliminary result, presented in Figure 6, shows that in principle arrays of differently coated sensors materials can be fabricated and read out simultaneously by using distributed fiber sensing. However, future research should focus on better suited polymers for chemical sensing. Such candidates could be metal organic framework (MOFs) [25], polymers of intrinsic microporosity (PIMS) [26], or electroactive polymers (EAPs) [27], which are advanced materials that exhibit interesting properties for strain-based sensing. Butterfly MOFs, for instance, have been shown to exhibit conformal switching upon exposure to electric fields [28]. PIMS have well-defined voids that increase the active surface area, yet have a relatively high Young’s modulus and the PIMS surface chemistry has shown to be made selective to certain chemicals. EAPs are driven by electric fields or coulomb forces, while ionic EAPs are based on the mobility of ions. They usually exhibit high mechanical densities and large actuation forces. Transfer methods or synthesis of these material right on the optical fibers will have to be established. Certainly, the fabrication methods will have to be adapted for each material.

## 4. Conclusions

We explored methods that integrate many different chemical sensing elements onto a single optical fiber. We demonstrated that it is possible to fabricate and read out the response from multiple different coatings. Stripping the original coating and recoating is a viable fabrication method for small fabrication batches. Fusion splicing of multiple fibers with different coatings (as shown in Section 2.2), however, would be the solution of choice if specific coatings were readily available commercially or were to be applied on long fiber segments. The preliminary results are proof of principle that such technology can enable a simultaneous readout of the response of differently coated segments of the fibers. Since many different sensors materials can be tested, a principal component analysis (PCA) [29] can be chosen for the data analysis. This can help to identify specific target chemicals or even detect the composition of chemical mixtures. Unlike many electronic- or microsystem-based sensors, optical fibers show a higher resistance to corrosion and can be nearly maintenance-free and fabricated at a lower cost compared to other type of sensors. Therefore, accelerated by a further reduction in the cost of optoelectronic components, fiber optical-based sensing is expected to expand into various fields ranging from the food industry, waste water monitoring, or medical applications.

## 5. Patents

Measurement method and sensor device for chemical detection using optical fibers, Patent filed with “State Office for Inventions and Trademarks”—Romania, nr. A/00744, 18 November 2020.

## Data Availability

Not applicable.
